# How often and to what extent do admitted COVID-19 patients have signs of cardiac injury?

**DOI:** 10.1007/s12471-021-01571-w

**Published:** 2021-04-16

**Authors:** M. A. W. Habets, H. N. Sturkenboom, R. A. Tio, E. Belfroid, J. Hoogervorst-Schilp, H. J. Siebelink, C. W. Jansen, P. C. Smits

**Affiliations:** 1 Department of Cardiology, Catharina Hospital, Eindhoven, The Netherlands; 2 Knowledge Institute of Medical Specialists, Dutch Association of Medical Specialists, Utrecht, The Netherlands; 3 Department of Cardiology, Leiden University Medical Center, Leiden, The Netherlands; 4 Netherlands Society of Cardiology, Utrecht, The Netherlands; 5 Department of Cardiology, Maasstad Hospital, Rotterdam, The Netherlands; 6 Netherlands Heart Registration, Utrecht, The Netherlands

**Keywords:** COVID-19, Myocardial injury, Cardiac troponin

## Abstract

**Background:**

COVID-19 can cause myocardial injury in a significant proportion of patients admitted to the hospital and seems to be associated with worse prognosis. The aim of this review was to study how often and to what extent COVID-19 causes myocardial injury and whether this is an important contributor to outcome with implications for management.

**Methods:**

A literature search was performed in Medline and Embase. Myocardial injury was defined as elevated cardiac troponin (cTn) levels with at least one value > 99th percentile of the upper reference limit. The primary outcome measure was mortality, whereas secondary outcome measures were intensive care unit (ICU) admission and length of hospital stay.

**Results:**

Four studies and one review were included. The presence of myocardial injury varied between 9.6 and 46.3%. Myocardial injury was associated with a higher mortality rate (risk ratio (RR) 5.54, 95% confidence interval (CI) 3.48–8.80) and more ICU admissions (RR 3.78, 95% CI 2.07–6.89). The results regarding length of hospital stay were inconclusive.

**Conclusion:**

Patients with myocardial injury might be classified as high-risk patients, with probably a higher mortality rate and a larger need for ICU admission. cTn levels can be used in risk stratification models and can indicate which patients potentially benefit from early medication administration. We recommend measuring cTn levels in all COVID-19 patients admitted to the hospital or who deteriorate during admission.

**Supplementary Information:**

The online version of this article (10.1007/s12471-021-01571-w) contains supplementary material, which is available to authorized users.

## Clinical question

*How often and to what extent is myocardial injury caused by COVID-19 and is myocardial injury an important contributor to outcome with implications for management?*

## Introduction

The paradigm that the presence of cardiovascular disease is a risk factor for developing severe coronavirus disease 2019 (COVID-19) and that COVID-19 can cause myocardial injury has recently been described. The presence of myocardial injury, defined as elevated cardiac troponin (cTn) levels, varies between 4–37% in studies coming from China [1–6].

The mechanism of myocardial injury in COVID-19 patients is as yet not well understood and might be multifactorial. One of the possible mechanisms is the emergence of viral myocarditis due to direct infection of the myocardial cells by binding of severe acute respiratory syndrome coronavirus 2 (SARS-CoV-2) to the angiotensin-converting enzyme 2 receptor, which is expressed in the myocardium. However, cTn release can also be secondary to myocardial ischaemic injury. SARS-CoV‑2 is thought to induce an acute systemic inflammation with cytokine release, which contributes to a prothrombogenic state and eventually plaque instability.

In addition to this type I myocardial infarction caused by atherosclerotic plaque disruption, COVID-19 can also induce type II myocardial infarction caused by an imbalance between oxygen demand and supply. The oxygen demand is increased by fever and tachycardia, while hypotension and pneumonitis-induced hypoxaemia decrease the oxygen supply. This imbalance can provoke ischaemia, even in patients with limited or no coronary artery atherosclerosis [7, 8]. Furthermore, Takotsubo syndrome (stress cardiomyopathy) has been described in COVID-19 patients and may be caused by emotional stress during the pandemic, together with physical triggers such as sepsis and hypoxaemia [9].

It is plausible that patients with COVID-19 and acute myocardial injury have a worse outcome than those without myocardial injury. This phenomenon has previously been described in patients with acute respiratory disease admitted to the intensive care unit (ICU): in these patients, elevated cTn levels were associated with higher mortality [10]. In accordance with influenza virus-infected patients, cardiac injury is also associated with higher mortality and longer ICU admission [11, 12].

However, the aforementioned studies are hampered by selection bias and a small sample size. Questions therefore remain about whether and to what extent COVID-19 causes myocardial damage and whether myocardial injury is an important contributor to outcome with implications for management, such as medication, diagnostic tests, cardiovascular imaging, long-term follow-up and, perhaps, situations where patient triage is needed.

## Methods

A review of the literature was performed to answer the following question: What are the occurrence, extent and outcome of cardiac injury in admitted patients with COVID-19? This question was structured in PICO format.

**P**opulation: Admitted COVID-19 patients
**I**ntervention: Presence of cardiac injury, defined as elevated cTn levels with at least one value > 99th percentile of upper reference limit (according to the Fourth Universal Definition of Myocardial Infarction)
**C**omparison: Admitted COVID-19 patients without cardiac injury
**O**utcome: Mortality, revascularisation, ICU admission, days on ventilation, length of hospital stay and need for intervention (percutaneous coronary intervention (PCI), coronary artery bypass grafting (CABG), implantable cardiac defibrillator (ICD) implantation, left and/or right ventricular assist device support)

### Relevant outcome measures

Mortality and revascularisation were considered crucial outcome measures, whereas ICU admission, days on ventilation, length of hospital stay and need for intervention were considered important outcome measures. A priori, the working group did not define minimal clinically relevant differences for the outcome measures.

### Search and select

**Fig. 1:**
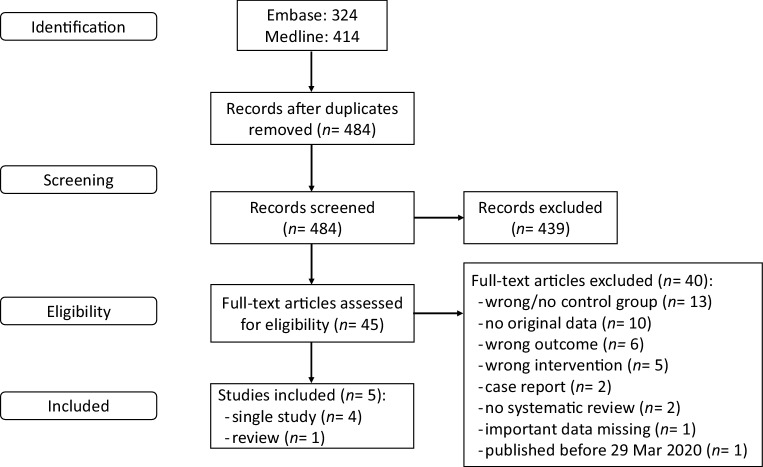
Study flow diagram

Fig. 1 shows the study flow diagram. The databases Medline (via Ovid) and Embase (www.embase.com) were searched using relevant search terms until 6 July 2020. The systematic literature search resulted in 484 hits (for details, see Table S1 in the Electronic Supplementary Material). The studies were independently screened by four authors.

Initially, 120 studies were included based on title and abstract screening. After a second assessment, in which the titles and abstracts of the 120 studies were assessed on inclusion criteria and outcomes, 45 studies were selected for full-text screening. Studies that described the underlying mechanism, included different outcome measures, did not define the intervention (elevated cTn level) correctly, did not contain original data, or were literature reviews but not systematic reviews were excluded.

After reading the full texts, 6 papers were included (1 systematic review, 5 single studies). Since the search of the systematic review was performed on 29 March 2020, all studies published before that date were excluded (1 study). In total, 5 papers (1 systematic review, 4 single studies) were included. The Grading Recommendations Assessment, Development and Evaluation (GRADE) system was used to assess the quality of evidence for the studies (www.gradeworkinggroup.org).

#### Description of studies

Important study characteristics and results are summarised in Tab. 1. The evidence table of all individual studies and the assessment of the risk of bias are shown in the Electronic Supplementary Material, in Tables S2 and S3, respectively.

**Table 1:** Study characteristics of included studies

Study (first author name, reference)	Study type	Number of patients	Country	Cardiac injury definition	Method	Outcome
Santoso [13]	Systematic review	2389 (13 studies) *Mortality:* 1550 (7 studies) *ICU admission:* 524 (3 studies)	Not reported, except: ‘Most of the studies are from China’	Hs-cTnI level > 99th percentile Time of measurement: not reported	Odds ratio meta-analysis (Mantel-Haenszel)	Mortality, ICU admission
Barman [14]	Multicentre retrospective study	607	Turkey	Hs-cTnI serum level > 99th percentile upper reference limit, regardless of new abnormalities on ECG Time of measurement: at hospital admission	Chi-square test to assess differences in categorical variables between groups, Student’s *t*-test or Mann-Whitney U test to compare unpaired samples as needed, Cox regression model	Mortality, ICU admission, length of hospital stay
Kuno [15]	Retrospective study	8438 5320 in whom cTnI was measured	USA	cTnI level elevation (defined as 99th percentile upper reference limit) Time of measurement: not reported	RR, stratification for age groups	Mortality
Lorente-Ros [16]	Matched retrospective cohort; after matching, adequate comparability was shown by a decrease of standardised differences to < 20% for all covariates	707	Spain	cTnI level > 99th percentile of healthy population Time of measurement: at hospital admission	Multivariate Cox proportional hazards regression models	Mortality, ICU admission, length of hospital stay
Wei [17]	Prospective assessment of medical records	101	China	Acute myocardial injury: hs-TnT level > institutional upper limit of normal (14 pg/mL) Time of measurement: at hospital admission	Student’s *t*-test or Mann-Whitney U test to compare mortality for elevated cTn levels, chi-square test	Mortality, ICU admission

It should be noted that the cardiac troponin (*cTn*) assays used in these studies differ in analytical characteristics, including their assessment of the upper reference limits, thereby limiting the direct comparability between studies

*hs-cTnI* high-sensitivity cardiac troponin I, *ICU* intensive care unit, *ECG* electrocardiography, *RR* risk ratio, *hs-TnT* high-sensitivity troponin T

The aim of the review by **Santoso** [13] was to explore the association between cardiac injury and mortality, need for ICU care, acute respiratory distress syndrome, and COVID-19 in patients with COVID-19 pneumonia. For this review, the authors searched for relevant articles in PubMed, Scopus, Europe PMC, ProQuest, and Cochrane CENTRAL databases. Search results were limited to the year ‘2020’. Articles other than original research, duplicate publication, and non-English articles were excluded. The search was finalised on 29 March 2020. A total of 13 studies were included. All studies were retrospective observational studies, but 4 were not peer-reviewed. Most of the included studies defined cardiac injury as high-sensitivity cardiac troponin I (hs-cTnI) level > 99th percentile although the troponin cut-off value was different in the included studies. Seven of the included studies reported on mortality and were included in a meta-analysis (risk ratio Mantel-Haenszel). Three studies were included in a meta-analysis of the relation between cardiac injury and ICU admission (risk ratio Mantel-Haenszel).

**Barman** [14] aimed to delineate the prognostic importance of the presence of concomitant cardiac injury for admission of patients with COVID-19. In this multicentre retrospective observational study, data of consecutive patients who were treated for COVID-19 between 20 March and 20 April 2020, were collected. Acute cardiac injury was defined as hs-cTnI serum levels > 99th percentile of the upper reference limit. In-hospital clinical outcome was compared between patients with and without cardiac injury. A total of 607 hospitalised patients with COVID-19 were included in the study. Cardiac injury was detected in 150 of them (24.7%).

**Kuno** [15] aimed to investigate whether cardiovascular disease or cardiac injury increases the risk of mechanical ventilation or mortality. Kuno retrospectively analysed a cohort of 8438 COVID-19 patients seen between 1 March and 22 April 2020. Of these patients, 4616 (54.7%) were admitted to hospital. Analysis was performed on 30 April 2020 and included patients who remained in hospital. Cardiac injury was defined as cTnI level elevation, which was defined as the 99th percentile of the upper reference limit. Cardiac injury was detected in 43.5% of the patients for whom cTnI measurements were available.

**Lorente-Ros** [16] studied the effect of myocardial injury assessment on risk stratification of COVID-19 patients. In this observational study, a matched cohort of 112 patients was created. After matching, an adequate comparability was shown by a decrease of the standardised differences to < 20% for all covariates. Mortality rate was compared between patients with and without cardiac injury. Cardiac injury was defined as cTnI levels > 99th percentile of a healthy population. Elevated cTnI levels were present in 20.9% of the total study population of 707 patients.

**Wei** [17] sought to characterise the prevalence and clinical implications of acute myocardial injury in a large cohort of patients with COVID-19. Data of 103 consecutive COVID-19 patients were collected between 16 January and 10 March 2020. Acute myocardial injury was defined as a high-sensitivity troponin T (hs-TnT) level > institutional upper limit of normal (14 pg/mL). Outcomes of interest included death, ICU admission, need for mechanical ventilation, treatment with vasoactive agents and classification of disease severity. Acute myocardial injury was present in 15.8% of the patients, nearly half of whom had a hs-TnT level 5‑fold the normal upper limit.

### Statistical analysis

Review Manager (version 5.4) was used to perform meta-analyses. Risk ratio (RR) with 95% confidence interval (CI) was calculated for all individual studies and used to calculate a pooled RR with 95% CI. Heterogeneity among studies was evaluated with the I^2^ test. Assessment of the risk of bias was based on the Quality in Prognostic Studies (QUIPS) tool [18, 19].

## Results

The included studies reported on mortality (Santoso, Barman, Kuno, Lorente-Ros, Wei), ICU admission (Santoso, Barman, Lorente-Ros, Wei) or length of hospital stay (Barman, Lorente-Ros). Ventilation was also an outcome measure in some studies (Kuno, Lorente-Ros, Wei); however, this was not defined as days on ventilation but as need for ventilation. None of the included studies mentioned revascularisation or another intervention (PCI, CABG, ICD implantation, or ventricular assist device support) as primary or secondary outcome. Therefore, we only report on mortality, ICU admission and length of hospital stay.

### Mortality

The systematic review by **Santoso** [13] described 7 studies in which the outcome measure mortality was studied, of which 4 were not peer-reviewed. The authors calculated a pooled RR of 7.95 (95% CI 5.12–12.34). The I^2^ was 65%, indicating possible substantial heterogeneity.

**Barman** [14], **Kuno** [15], **Lorente-Ros** [16] and **Wei** [17] also studied mortality in relation to cardiac injury. Barman and Lorente-Ros performed a univariate and multivariate regression analysis. In the study by **Barman** [14], the univariate analysis (30 days) resulted in an odds ratio (OR) of 7.97 (95% CI 5.03–12.64, *p* < 0.001). The multivariate regression model (30 days) resulted in an OR of 10.58 (95% CI 2.42–46.27, *p* < 0.001). In the multivariate model, age, sex, uric acid, hypertension, diabetes mellitus, coronary artery disease, smoking, chronic obstructive pulmonary disease, creatinine, glucose, C‑reactive protein (CRP) and D‑dimer ≥ 500 ng/mL were taken into account, in addition to cardiac injury.

In the study by **Lorente-Ros** [16], in the matched cohort, all-cause mortality within 30 days was higher in patients with cTnI level elevation than in those with lower levels (41.1% vs 23.2%; *p* = 0.005). The univariate regression model (30 days) resulted in a hazard ratio (HR) of 4.355 (95% CI 3.112–6.093, *p* < 0.001). The multivariate regression model (30 days) resulted in an HR of 1.716 (95% CI 1.182–2.492, *p* = 0.005). In the multivariate model, sex, age, hypertension, renin-angiotensin-aldosterone system inhibitor use, haematocrit, creatinine, D‑dimer, CRP and Charlson Comorbidity Index were taken into account, in addition to cardiac injury.

The study by **Kuno** [15] showed an RR of 5.07 (95% CI 4.45–5.76) for mortality. In the study by **Wei** [17], the log hs-TnT level was associated with disease severity (OR 6.63, 95% CI 2.24–19.65), and all three deaths occurred in patients with acute myocardial injury.

**Fig. 2:**
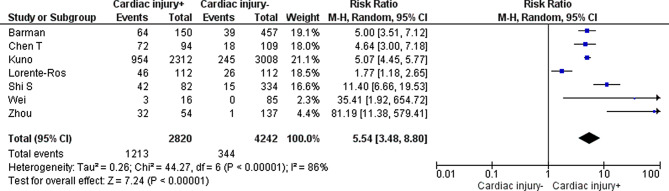
Pooled risk ratio of cardiac injury in relation to mortality (*M‑H* Mantel-Haenszel, *CI* confidence interval)

We calculated a pooled RR in which only the peer-reviewed studies from the systematic review by **Santoso** [13] and the results reported by **Barman** [14], **Kuno** [15], **Lorente-Ros** [16] and **Wei** [17] were included (Fig. 2). The pooled RR of COVID-19 patients with cardiac injury in relation to mortality was 5.54 (95% CI 3.48–8.80). The I^2^ was 86%, indicating substantial heterogeneity among the included studies.

The level of evidence was downgraded to ‘low’ because of risk of bias (retrospective design, often no correction for confounders). For more details, see Table S3 in the Electronic Supplementary Material.

### ICU admission

In the systematic review by **Santoso** [13], there were 3 studies assessing the outcome measure ICU admission, which were included in a meta-analysis. Of the individual studies, **Barman** [14], **Lorente-Ros** [16] and **Wei** [17] assessed ICU admission.

For ICU admission, the systematic review by **Santoso** [13] showed a pooled RR of 7.94 (95% CI 1.51–41.78). The studies by **Barman** [14] (72% vs 19%; *p* < 0.001) and **Wei** [17] (62.5% vs 24.7%; *p* = 0.003) showed a significant difference between COVID-19 patients with and without cardiac injury. **Lorente-Ros** [16] concluded there was no significant difference between both groups (6.3% vs 4.3%; *p* = 0.527). However, the number of patients requiring ICU admission in this study was very small (7 vs 5), which might have influenced the effect.

**Fig. 3:**
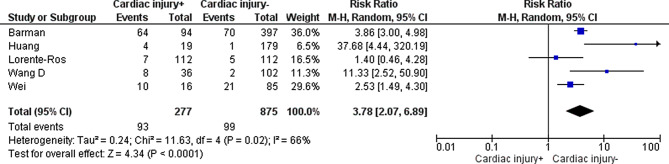
Pooled risk ratio of cardiac injury in relation to intensive care unit admission (*M‑H* Mantel-Haenszel, *CI* confidence interval)

A pooled RR was calculated including only the peer-reviewed studies from the systematic review by **Santoso** [13] and the individual studies that assessed ICU admission (Fig. 3). The pooled RR of COVID-19 patients with cardiac injury in relation to ICU admission was 3.78 (95% CI 2.07–6.89). The I^2^ was 66%, indicating substantial heterogeneity among the studies.

The level of evidence was downgraded to ‘very low’ because of risk of bias (retrospective design, often no correction for confounders) and indirectness (none of the studies performed in the Netherlands, possibly different criteria for ICU admission in each country, ICU admission possibly dependent on ICU capacity) (see Table S3 in the Electronic Supplementary Material for more details).

### Length of hospital stay

**Barman** [14] reported a significant difference in length of hospital stay between patients with cardiac injury and those without cardiac injury (11 days, range 5–16 vs 9 days, range 4–11, *p* = 0.002), whereas **Lorente-Ros** [16] did not detect a significant difference (11 days, interquartile range (IQR) 6–17 vs 9 days, IQR 5–13, *p* = 0.934).

The level of evidence was downgraded to ‘very low’ because of inconsistency (different conclusion for the two studies assessing length of hospital stay), indirectness (studies performed in different European countries, possibly different criteria for hospital discharge in each country) and imprecision (only two studies included, low number of patients, follow-up length of stay and number of patients lost to follow-up unclear) (see Table S3 in the Electronic Supplementary Material for more details).

## Conclusion

In this literature review on COVID-19 patients, the presence of myocardial injury (defined as elevated cTn levels) varied between 9.6 and 46.3%. Furthermore, myocardial injury was associated with a worse prognosis, a higher mortality rate (RR 5.54, 95% CI 3.48–8.80) and more ICU admissions (RR 3.78, 95% CI 2.07–6.89). Despite the low GRADE level of evidence, caused by the retrospective design and substantial heterogeneity among the included studies, all studies showed a worse outcome in patients with myocardial injury. Therefore, we recommend measuring cTn levels in all COVID-19 patients who are admitted to the emergency department and in those who deteriorate during admission. Patients with elevated cTn levels might be classified as high-risk patients, with probably a larger need for hospitalisation, ICU admission and additional cardiac diagnostics, and a higher mortality rate. cTn levels can be used in risk stratification models and can indicate which patients may benefit from early medication administration and cardiac imaging.

### Mortality (crucial)

Cardiac injury (defined as cTn level elevation > 99th percentile) in COVID-19 patients could be associated with a higher mortality risk. *Sources: Santoso, Lorente-Ros, Barman, Wei ***(low GRADE level)**.

### ICU admission (important)

We are unsure if cardiac injury (defined as cTn level elevation > 99th percentile) in COVID-19 patients is associated with ICU admission. *Sources: Santoso, Lorente-Ros, Barman, Wei* **(very low GRADE level)**.

### Length of hospital stay (important)

We are unsure if cardiac injury (defined as cTn level elevation > 99th percentile) in COVID-19 patients is associated with the number of admission days in hospital. *Sources: Barman, Lorente-Ros* **(very low GRADE level)**.

## Discussion

The current review demonstrated that ICU admissions were more frequently required and the mortality rate was higher in COVID-19 patients with myocardial injury. This clinically relevant effect involves a major patient population. cTn levels are easily measured in every hospital, the costs are low and there are no drawbacks for the patient as the test can be included in routine laboratory testing during hospital admission.

To distinguish between ischaemic and non-ischaemic myocardial injury, it is important to perform a specific cardiovascular history and examination, electrocardiography (ECG), cardiac imaging with echocardiography, and sometimes cardiac magnetic resonance imaging (MRI) or coronary angiography. This does not only provide information on the underlying mechanism of the myocardial injury, but also on the direct effect of myocardial injury on the left and right ventricular function and coronary arteries. This knowledge may have therapeutic implications, such as need for heart failure medication or revascularisation. Elevated cTn levels, together with other laboratory values and patient characteristics, can also be used in risk stratification models for early triage of high-risk COVID-19 patients and can aid in medical decision-making, for example regarding early medication administration [20, 21].

In a recently published German study, 100 COVID-19 patients underwent cardiac MRI within 3 months of diagnosis [22]. Of these patients, 78% had an abnormal MRI scan, 60% had signs of ongoing myocardial inflammation and 12% had an ischaemic-type pattern of myocardial late gadolinium enhancement. This may indicate that viral myocarditis is one of the main underlying mechanisms of cardiac injury. However, a recently published autopsy study showed contradictory results. In this study, 39 deceased patients with COVID-19 were autopsied. None of them were diagnosed as having clinically fulminant myocarditis. In 61% of the autopsies, SARS-CoV‑2 RNA was present in the myocardium, and in 41%, the viral load was > 1000 copies (which is deemed to be clinically significant). Still, none of the deceased patients met the Dallas criteria for myocarditis, because no massive cell infiltrates or necrosis could be documented. The most likely localisation of SARS-CoV‑2 was not the cardiomyocytes but the interstitial cells. These data indicate that the presence of SARS-CoV‑2 in cardiac tissue does not necessarily cause an inflammatory reaction [23].

In an Israeli study, 100 COVID-19 patients underwent transthoracic echocardiography within 24 h of hospital admission [24]. Right ventricular dilatation and dysfunction was the most common cardiac pathology (39% of the patients), followed by left ventricular diastolic dysfunction (16%) and left ventricular systolic dysfunction (10%). Patients with elevated cTn levels (20% of total population) had worse right ventricular function and left ventricular diastolic function compared with patients with normal cTn levels. A previous study in ICU patients with severe sepsis also showed that isolated left ventricular diastolic dysfunction is more common than systolic dysfunction and that diastolic dysfunction is a stronger predictor of mortality [25].

As elevated cTn levels enhance the need for cardiac imaging in selected patients, medical costs and waiting lists will increase during admission. However, cTn levels can aid in selecting patients who benefit from early interventions such as revascularisation and medication. In the long term, this can lead to health benefits and shorten or prevent hospital admissions.

In most included studies, cTn levels were measured once, at hospital admission. However, the time of measurement was not always reported, and it is possible that the cTn level was only measured when the clinical situation deteriorated. This causes selection bias. For prognostic reasons, it is important to know the maximum cTn levels. It is possible that patients with chronically elevated cTn levels were also included in the selected studies. cTn levels can be chronically elevated in several chronic diseases, such as heart failure, diabetes mellitus, pulmonary arterial hypertension and kidney disease [26]. These chronic comorbidities are also known risk factors for severe and complicated COVID-19 [27, 28]. The question is whether the higher mortality rate and larger need for ICU admission in our patient group can be fully attributed to new cardiac injury caused by COVID-19 or whether the elevated cTn levels are a result of these chronic diseases with consequently higher mortality rates. It is important to do serial cTn testing to detect a rise and fall in cTn levels, which discriminates acute myocardial injury from chronically elevated cTn levels.

Most of the presented studies are from outside Europe (the majority is from China) and from places were COVID-19 had a high impact on medical resources. This may have led to delayed hospital admissions and management and may have increased the degree of cardiac injury [17]. It is unclear whether the results of this review also apply to the situation in the Netherlands. In the Dutch healthcare system, the first and second echelons are well intertwined and the driving times of ambulances to the emergency departments are short. This system possibly reduces delays in hospital admission and the degree of cardiac injury. The results of the Dutch multicentre CAPACITY COVID Registry are expected soon and will show whether the degree of cardiac injury and outcome of COVID-19 patients in the Netherlands are comparable with the results of this review.

### Recommendations

Measurement of cTn levels in COVID-19 patients may be highly valuable for diagnostic and prognostic purposes. Because of the low costs and the fact that the test can be included in routine laboratory testing during hospital admission, we advise to determine cTn levels in all hospitalised COVID-19 patients. Serial cTn testing is important to detect a rise and fall to distinguish between chronically elevated cTn levels and new myocardial injury (see Box 1).

#### Box 1 Recommendations

- Determine cardiac biomarkers (cTn, creatine kinase–myocardial band (CK-MB) and N‑terminal pro-brain natriuretic peptide (NT-pro-BNP)) in all hospitalised COVID-19 patients, and do serial testing in selected patients based on clinical grounds.
- Perform cardiac history, ECG and imaging during or after hospital admission in selected patients based on aforementioned serial laboratory tests

### Gaps in evidence

Several questions do remain. For example, what is the most common underlying mechanism of myocardial injury in COVID-19 patients? Does standardised cardiac imaging with echocardiography, cardiac MRI or coronary angiography have therapeutic implications for COVID-19 patients? And what is the prognostic impact of myocardial injury on the short-term (days to weeks) and long-term outcomes (months to years) of COVID-19 patients?

## Supplementary Information

Table S1 Literature search strategy

Table S2 Evidence tables

Table S3 Risk of bias
