# Supplementary material for: How often and to what extent do admitted COVID-19 patients have signs of cardiac injury?
Source: Neth Heart J. 2021 Apr 16;29(Suppl 1):5–12. doi: 10.1007/s12471-021-01571-w (PMC8050638; doi:10.1007/s12471-021-01571-w)
Supplement: Supplementary file 1 — Table S1 Literature search strategy [file 12471_2021_1571_MOESM1_ESM.docx]

**Literature search strategy**

**Ovid/Medline**

1 exp Myocardial Ischemia/ or ((cardia* or heart or myocard* or coronary or endocard* or subendocard*) adj3 (attack or infarction or ischemi* or ischaemi* or anoxia or hypoxia or lesion or injury or damage or trauma)).ti,ab,kf. (535266)

2 ((exp Coronavirus/ or Coronavirus Infections/ or pneumonia virus*.ti,ab,kf. or cov.ti,ab,kf.) and ((outbreak or wuhan).ti,ab,kf. or novel.af. or '19'.ti,ab,kf. or '2019'.ti,ab,kf. or epidem*.af. or epidemy.af. or epidemic*.af. or pandem*.af. or new.ti,ab,kf.)) or (coronavirus* or 'corona virus*' or ncov or '2019ncov' or 'covid19' or "covid 19" or "sars cov 2" or 'sars2' or "ncov 2019" or "sars coronavirus 2" or "sars corona virus 2" or "severe acute respiratory syndrome cov 2" or "severe acute respiratory syndrome cov2" or "severe acute respiratory syndrome cov*").ti,ab,kf. (43132)

3 limit 2 to yr="2019 -Current" (31021)

4 1 and 3 (414)

5 4 not ((exp animals/ or exp models, animal/) not humans/) not (letter/ or comment/ or editorial/) (356)

6 (meta-analysis/ or meta-analysis as topic/ or (meta adj analy$).tw. or (systematic*or literature adj2 review$1).tw. or (systematic adj overview$1).tw. or exp "Review Literature as Topic"/ or cochrane.ab. or cochrane.jw. or embase.ab. or medline.ab. or (psychlit or psyclit).ab. or (cinahl or cinhal).ab. or cancerlit.ab. or ((selection criteria or data extraction).ab. and "review"/)) not (Comment/ or Editorial/ or Letter/ or (animals/ not humans/)) (293781)

7 5 and 6 (14) SR

8 4 not 7 (400) Overige

**Embase**

| No. | Query | Results |
| --- | --- | --- |
| #7 | #3 NOT #6 Overige | **305** |
| #6 | #4 AND #5 SR | **19** |
| #5 | ('meta analysis'/de OR 'meta analysis (topic)'/exp OR cochrane:ab OR embase:ab OR psycinfo:ab OR cinahl:ab OR medline:ab OR ((systematic NEAR/1 (review OR overview)):ab,ti) OR ((meta NEAR/1 analy*):ab,ti) OR metaanalys*:ab,ti OR 'data extraction':ab OR cochrane:jt OR 'systematic review'/de) NOT (('animal experiment'/exp OR 'animal model'/exp OR 'nonhuman'/exp) NOT 'human'/exp) | **524957** |
| #4 | #3 NOT ('conference abstract'/it OR 'editorial'/it OR 'letter'/it OR 'note'/it) NOT (('animal experiment'/exp OR 'animal model'/exp OR 'nonhuman'/exp) NOT 'human'/exp) | **324** |
| #3 | #1 AND #2 | **392** |
| #2 | ('coronavirus disease 2019'/exp OR (('coronavirinae'/exp OR 'coronavirus infection'/de OR coronavirus*:ti,ab,kw OR 'corona virus*':ti,ab,kw OR 'pneumonia virus*':ti,ab,kw OR cov:ti,ab,kw OR ncov:ti,ab,kw) AND (outbreak:ti,ab,kw OR wuhan:ti,ab,kw)) OR covid19:ti,ab,kw OR 'covid 19':ti,ab,kw OR ((coronavirus*:ti,ab,kw OR 'corona virus*':ti,ab,kw) AND 2019:ti,ab,kw) OR 'sars cov 2':ti,ab,kw OR sars2:ti,ab,kw OR 'coronavirus*':ti,ab,kw OR 'corona virus*':ti,ab,kw OR 'ncov 2019':ti,ab,kw OR ncov:ti,ab,kw OR 'sars coronavirus 2':ti,ab,kw OR 'sars corona virus 2':ti,ab,kw OR 'severe acute respiratory syndrome cov 2':ti,ab,kw OR 'severe acute respiratory syndrome cov2':ti,ab,kw) AND [2019-2020]/py | **24590** |
| #1 | 'heart muscle injury'/exp OR 'heart muscle ischemia'/exp OR 'heart infarction'/exp OR (((cardia* OR heart OR myocard* OR coronary OR endocard* OR subendocard*) NEAR/3 (attack OR infarction OR ischemi* OR ischaemi* OR anoxia OR hypoxia OR lesion OR injury OR damage OR trauma)):ti,ab,kw) | **601459** |
