# Supplementary material for: How often and to what extent do admitted COVID-19 patients have signs of cardiac injury?
Source: Neth Heart J. 2021 Apr 16;29(Suppl 1):5–12. doi: 10.1007/s12471-021-01571-w (PMC8050638; doi:10.1007/s12471-021-01571-w)
Supplement: Supplementary file 2 — Table S2 Evidence tables [file 12471_2021_1571_MOESM2_ESM.docx]

**Evidence tables**

*Evidence table studies included in systematic review Santoso, 2020*

| **Study reference** | **Study characteristics** | **Patient characteristics** | **Prognostic factor (cardiac injury definition)** | **Follow-up** | **Outcome measures and effect size** | **Comments** |
| --- | --- | --- | --- | --- | --- | --- |
| Santoso, 2020 | SR and meta-analysis of cohort studies  *Literature search up to 29 March 2020*  **A**: Chen T, 2020 (mortality)  **B**: Li K, 2020 (mortality) [not peer reviewed]  **C**: Luo XM, 2020 (mortality) [not peer reviewed]  **D**: Shi S, 2020 (mortality)  **E**: Wu C, 2020 (mortality, IC admission) [not peer reviewed]  **F**: Zhang F, 2020 (mortality) [not peer reviewed]  **G**: Zhou 2020 (mortality)  **H**:Wang D, 2020 (IC admission)  **I**: Huang, 2020 (IC admission)  **J**: Hu L, 2020  **K**: Hu B, 2020  **L**: Zhao W, 2020  **M**: Zhang Guqin, 2020  Study design: all studies are observational retrospective studies  Setting and Country: Not available per study, most of the studies are from China  Source of funding: Not reported | Inclusion criteria SR: all research articles in adult patients diagnosed with COVID-19 with information on hs-cTnl, cardiac injury, and clinical grouping or outcome of the clinically validated definition of mortality, the need for ICU care, acute respiratory distress syndrome (ARDS), or severe COVID-19  Exclusion criteria SR: articles other than original research (e.g., case report or series, review articles, letters to editor, editorials or commentaries), duplicate publication, and non-English articles.  *13 studies included (7 report on mortality and 3 on IC admission)*  Important patient characteristics at baseline:  N, mean age deceased vs mean age survivors  **A**: N=799, analysis based on N=274, 68y(†) vs 51y  **B:** N=32, 69y(†) vs 51y  **C**: N=403, 71y(†) vs 59y  **D**: N=416, N/A  **E**: N=188, N/A  **F**: N=48, 78.65y(†) vs 66.16y  **G**: N=191, 69y(†) vs 52y  **H**: N=138, 66y (†) vs 51y  **I**: N=41, 49y(†) vs 49y  **J**: N=323, 65y(†) vs 56y  **K**: N=36, 66.5y(†) vs 56y  **L**: N=78, 69y(†) vs 45y  **M**: N=221, 62(†) vs 51y  *Sex (% male, deceased vs survivors*)*:*  **A**: 73%(†) vs 55%  **B**: 73%(†) vs 22%  **C**: 57%(†) vs 44.9%  **D**: N/A  **E**: N/A  **F**: 70.6%(†) vs 67.7%  **G**: 70%(†) vs 59%  **H**: 61.1%(†) vs 52%  **I**: 85%(†) vs 68%  **J**: 52.9%(†) vs 49.7%  **K**: 68.8%(†) vs 65% **L**: 55%(†) vs 40.4%  **M**: 63.6%(†) vs 44%  Groups comparable at baseline Patients that deceased are older and more frequently male then the survivors. | **A**: hs-cTnl above 99th percentile  **B**: Unspecified  **C**: Unspecified  **D**: hs-cTnl above 99th  percentile  **E**: Unspecified  **F**: hs-cTnl above 99th  percentile  **G**: hs-cTnl above 99th  percentile  **H**: hs-cTnl above 99th  percentile  **I**: hs-cTnl above 99th  percentile  **J**: Unspecified  **K**: Unspecified  **L**: Unspecified  **M**: hs-cTnl above 99th percentile | End-point of follow-up:  **A**: Max 46 days (patients admitted from 13 January 2020 to 28 February 2020)  **B**: Max 34 days (Patients admitted from January 31 to March 5, 2020)  **C**: Max 26 days (patients admitted from Jan 30 to Feb 25, 2020)  **D**: Max 26 days (patients admitted from January 20, 2020, to February 10, final date of follow up February 15, 2020)  **E**: Max 48 days (patients admitted from December 25, 2019 to January 27, 2020, follow-up complete on February 11, 2020)  **F**: Max 52 days (patients admitted from December 25, 2019 to February 15, 2020)  **G**: Max 33 days (patients admitted between December 29 and Janaury 31, 2020)  **H**: Max 33 days (patients admitted from January 1 to January 28, 2020, follow up until February 3^,^ 2020)  **I**: N/A (patients admitted from December 16, 2019, to January 2, 2020)  **J**: average observation period 28 days (20-47 days) (patients enrolled from January 8 to February 20, 2020, follow up until March 10, 2020)  **K**: N/A (patients admitted from January 8 to February 9, 2020)  **L**: Max 39 days (patients admitted from January 21 to February 8, 2020, follow up until February 29, 2020)  **M**: Max 44 days (patients admitted from January 2, 2020 to February 10, 2020, follow up until February 15, 2020)  For how many participants were no complete outcome data available?  **A: 525**  **B**: 5  **C**: 0  **D**: N/A  **E**: 0  **F**: 2  **G**: 0  **H**: 0  **I**: 0  J: 0  K: 14  L: 41  M: 168 | Outcome measure Mortality  Effect measure: RR [95% CI]  **A**: 4.64 [3.00, 7.18]  **B**: 6.69 [2.61, 17.17] [not peer reviewed]  **C**: 10.94 [6.83, 17.52] [not peer reviewed]  **D**: 11.40 [ 6.66, 19.53]  **E**: 5.25 [2.90, 9.50] [not peer reviewed]  **F**: 6.08 [1.93, 19.13] [not peer reviewed]  **G**: 81.19 [11.38, 579.41]  Pooled risk ratio 7.95 [5.12 – 12.34]  Heterogeneity (I^2^): 65%  Outcome measure IC admission  Effect measure: RR [95% CI]  **E**: 2.39 [1.50, 3.80] [not peer reviewed]  **H**: 11.33 [2.52, 50.90]  **I**: 37.68 [4.44, 320.19]  Pooled risk ratio 7.94 [1.51 – 41.78]  Heterogeneity (I^2^): 79% | Most of the included studies are from China, are pre-prints and have a small number of included participants. Patients are included in the early days of the COVID pandemic (most of them enrolled in January and February 2020). Patients that deceased are older and more frequently male then the survivors. The effect is measured in RR, so a correction for this confounding factors was not performed.  A sensitivity analysis by leave-one-out was performed to single out heterogeneity. Sensitivity analysis showed that heterogeneity for mortality outcomes could be reduced by removal of G: Zhou 2014 et al. study (RR 7.22 [4.97, 10.47], p < 0.001: I^2^: 54%, p = 0.05).  The removal of E: Wu et al. reduced heterogeneity for the need for ICU care (RR 16.85 [4.93, 57.62],  p < 0.001; I^2^: 0%, p = 0.36) |

*Evidence table single studies*

| **Study reference** | **Study characteristics** | **Patient characteristics** | **Prognostic factor(s)** | **Follow-up** | **Outcome** |  |
| --- | --- | --- | --- | --- | --- | --- |
| Barman, 2020 | Type of study: multi-center retrospective study  Setting: COVID-19 patients who were hospitalized in three government hospitals  Country: Turkey  Source of funding:  N/A | Inclusion criteria: consecutive COVID-19 patients who were hospitalized in three government hospitals in Istanbul, Turkey between March 20, 2020 and April 20, 2020  Exclusion criteria: Patients < 18 y, with concurrent ST-segment elevation myocardial infarction, with history of advanced kidney failure [estimated glomerular filtration rate (eGFR) <30 ml/min] or hemodialysis and patients with missing laboratory parameters on admission including hs-TnI, and creatine kinase myocardial band (CK-MB)  N= 607 Cardiac injury+ N= 150 Cardiac injury– N = 457  Mean age ± SD: Cardiac injury+: 66.0y ± 14.5 Cardiac injury-: 55.3y ± 15.2  Sex(%male):  Cardiac injury+: 54% Cardiac injury-: 52% | high sensitivity cardiac troponin I serum levels above the 99th percentile upper reference limit, regardless of new abnormalities in ECG  Moment of measurement: at hospital admission | patients were hospitalized between March 20, 2020 and April 20, 2020, follow up until April, 20 2020  For how many participants were no complete outcome data available?  N (%): N/A  Reasons for incomplete outcome data described?  No | Mortality Cardiac injury+ = 64 (42%) Cardiac injury– = 39 (8%) P<0.001  Univariable regression model (30 days) OR 7.97 [5.03–12.64] P <0.001  Multivariable regression model (30 days) OR 10.58 [2.42–46.27] P<0.001  Cardiac injury was found to be a predictor of mortality.  Subgroup analysis  When patients with previous CAD were excluded from analyses, presence of cardiac injury was still an independent predictor of mortaliy (OR 2.52, 95% CI 1.17–5.45; P = 0.018)  IC admission Cardiac injury+ N= 108 (72%) Cardiac injury– N= 87 (19%) P<0.001  Patients with cardiac injury were more frequently admitted to the IC then patients without cardiac injury.  Hospital duration (days) Cardiac injury+: 12 (5–14) days Cardiac injury–: 9 (4–12) days P<0.001  Patients with cardiac injury spent more days in the hospital then patients without cardiac injury. | Student’s t-test or Mann–Whitney U test, Cox regression model |
| Kuno, 2020 | Type of study: Retrospective cohort  Setting: 7 hospitals with more than 3800 beds and more that 410 ambulatory practices across metropolitan New York  Country:  US  Source of funding:  No extramural funding | Inclusion criteria: N/A  Exclusion criteria: N/A  N = 8438 5320 used for analysis (troponin measured)  Median age ± SD:  59 [43, 71]  Sex:  53.9% male | Cardiac injury was defined as troponin I elevation which was defined as 99th percentile upper reference limit  Moment of measurement: not reported | Endpoint of follow-up: Patients admitted from March 1 to April 22, 2020, follow up until April 30, 2020)  For how many participants were no complete outcome data available?  N (%): N/A  Reasons for incomplete outcome data described? No | Mortality Cardiac injury+: 41.3% (954/2312)  Cardiac injury –: 8.1% (245/3008)  RR 5.07 (4.45-5.76)  Patients with cardiac injury have an increased risk of mortality | RR |
| Lorente-Ros, 2020 | Type of study: Matched retrospective cohort  Setting:  a large tertiary hospital  Country:  Spain  Source of funding:  N/A | Inclusion criteria: patients aged 18 years and older admitted to a large tertiary hospital with COVID-19 infection were retrospectively included with prospective follow-up.  Exclusion criteria: primary cardiac presentation, i.e. type 1 myocardial infarction  N=707 Cardiac injury+ n = 112 Cardiac injury- n=112  Mean age ± SD: 66.76 ± 15.7 years  Sex:  63% male | cTnI levels greater than the 99th percentile of a healthy population  Moment of measurement: at hospital admission | Endpoint of follow-up:Patients admitted from March 18 to March 23, 2020 All patients were followed for 1 month  For how many participants were no complete outcome data available?  For the total group the outcome data of 66 (out of 707) remained hospitalized after 1 month. For the matched cohort the information is not available.  Reasons for incomplete outcome data described? N/A | Mortality Cardiac injury+ N= 46 (41.1%)  Cardiac injury- N= 26 (23.2%) P=0.005  All-cause mortality within 30 days was higher in those with cTnI elevation  Univariable regression model (30 days) Hazard Ratio 4.355 (3.112–6.093) P< 0.001  Multivariable regression model (30 days) Hazard Ratio 1.716 (1.182–2.492) P= 0.005  cTnI elevation was independently associated with a higher risk of all-cause mortality within 30 days. Age, CRP and creatinine on admission were also independent prognostic factors.  IC admission Cardiac injury+ N=7 (6.3%) Cardiac injury- N= 5 (4.5%) P= 0.527  There is no difference in patient with and without cardiac injury regarding IC-admission  Hospital duration (median days) Cardiac injury+: 11 (6 to 17) days Cardiac injury-: 9 (5 to 13) days P= 0.934  There is no difference in patients with and without cardiac injury regarding hospital duration | Multivariate Cox proportional hazards regression models, comparing means |
| Wei, 2020 | Type of study: Propspective assessment of medical records  Setting: laboratory-confirmed SARS-CoV-2 infection admitted to the Public Health Clinical Centre of Chengdu and West China Hospital, Sichuan University  Country:  China  Source of funding:  none declared | Inclusion criteria: N/A  Exclusion criteria: N/A  N=101  hs-TnT≤14pg/mL n=85 hs-TnT>14pg/mL n=16  Mean age ± SD: Total 49 (34–62)y hs-TnT≤14pg/mL: 47 (33–55)y hs-TnT>14pg/mL : 67 (61.0–80.5)y P<0.001  Sex: % M  Total: 53.5% male hs-TnT≤14pg/mL: 55.3% male hs-TnT>14pg/mL : 43.8% male P= 0.401 | Acute myocardial injury was defined by an hs-TnT value greater than the institutional upper limit of normal (14pg/mL)  Moment of measurement: at hospital admission | Endpoint of follow-up: Patients admitted between between January 16, 2020, and March 10, 2020  For how many participants were no complete outcome data available?  N/A  Reasons for incomplete outcome data described? N/A | Mortality hs-TnT≤14pg/mL 0 death (0%) hs-TnT>14pg/mL 3 death (18.8%) P<0.001  IC admission hs-TnT≤14pg/mL 21 IC admissions (24.7%) hs-TnT>14pg/mL 10 IC admissions (62.5%) P=0.003  Patients with acute myocardial injury were more likely to require admission to ICU | Multivariate analysis |
