# Supplementary material for: How often and to what extent do admitted COVID-19 patients have signs of cardiac injury?
Source: Neth Heart J. 2021 Apr 16;29(Suppl 1):5–12. doi: 10.1007/s12471-021-01571-w (PMC8050638; doi:10.1007/s12471-021-01571-w)
Supplement: Supplementary file 3 — Table S3 Risk of bias [file 12471_2021_1571_MOESM3_ESM.docx]

**Risk of bias**

*Table of quality assessment – prognostic factor (PF) studies (studies included in systematic review Santoso, 2020)

Based on: QUIPS^A^ (Haydn, 2006; Haydn 2013)*

| **Study reference**  (first author, year of publication) | **Study participation^1^**  Study sample represents the population of interest on key characteristics?  (high/moderate/low risk of selection bias) | **Study Attrition^2^**  Loss to follow-up not associated with key characteristics (i.e., the study data adequately represent the sample)?  (high/moderate/low risk of attrition bias) | **Prognostic factor measurement^3^**  Was the PF of interest defined and adequately measured?  (high/moderate/low risk of measurement bias related to PF) | **Outcome measurement^3^**  Was the outcome of interest defined and adequately measured?  (high/moderate/low risk of measurement bias related to outcome) | **Study confounding^4^**  Important potential confounders are appropriately accounted for?  (high/moderate/low risk of bias due to confounding) | **Statistical Analysis and Reporting^6^**  Statistical analysis appropriate for the design of the study?  (high/moderate/low risk of bias due to statistical analysis) | **Peer reviewed** |
| --- | --- | --- | --- | --- | --- | --- | --- |
| **A**: Chen T, 2020 | Low (all patients diagnosed with Covid-19) | Low (for the analysis only the data of patients that died or were discharged from the hospital were included) | Moderate risk (hs-cTnl>15.6 pg/mL) | low | x | low | Yes |
| **B**: Li K, 2020 (mortality) | Low (all patients diagnosed with Covid-19) | Moderate (5 patients were still hospitalized at the moment of analysis) | Low risk (hs-cTnl 34.2pg/mL) | low | x | low | No |
| **C**: Luo XM, 2020 (mortality) | Low (all patients diagnosed with Covid-19) | Low (for the analysis data of patients that died or were discharged from the hospital were included) | Low risk (hs-cTnl>40 pg/mL) | low | x | low | No  Santoso also included incorrect results from this study. Should be:  Mortality  cardiac injury+: 47/96 (49.0%)  recovered: CI+ 18/208 |
| **D**: Shi S, 2020 (mortality) | Low (all patients diagnosed with Covid-19) | Moderate (319 patients remained in the hospital at the time of analysis) | Low risk (cardiac injury was defined as blood levels of cardiac biomarkers (hs-TNI) above the 99thpercentile upper reference limit, regardless of new abnormalities in electrocardiography and echocardiography) | low | x | low | Yes |
| **E**: Wu C, 2020 (mortality, IC admission) | Low (all patients diagnosed with Covid-19) | Low (all patients died or were discharged at the moment of analysis) | High risk (hs-cTnl≥ 6.126 pg/mL) | low | x | low | No |
| **F**: Zhang F, 2020 (mortality) | low (patients were diagnosed or suspected of Covid-19) | Low (all patients died or were discharged at the moment of analysis) | Low risk (hs-cTnI) were above the 99th percentile upper  reference limit (0.026ug/L) | low | x | low | No |
| **G**: Zhou 2020(mortality) | Low (all patients diagnosed with Covid-19) | Low (all patients died or were discharged at the moment of analysis) | Low (Acute cardiac injury was diagnosed if serum levels of cardiac biomarkers (eg, highsensitivity cardiac troponin I) were above the 99^th^ percentile upper reference limit, (≥28 pg/mL) or if new abnormalities were shown in electrocardiography and echocardiography) | Low | X | low | Yes |
| **H**:Wang D, 2020 (IC admission) | Low (all patients diagnosed with Covid-19) | Moderate (some patients remained hospitalized at the moment of analysis, number is unknown) | Low (hs-cTn were above the 99th percentile upper reference limit ≥26.2 pg/mL or new abnormalities were shown in electrocardiography and echocardiography) | low | X | low | Yes |
| **I**: Huang, 2020 (IC admission) | Low (all patients diagnosed with Covid-19) | Moderate (7 patients still hospitalized at the time of analysis) | Low (cardiac injury was diagnosed if serum levels of cardiac biomarkers (eg, troponin I) were above the 99th percentile upper reference limit >28 pg/mL, or new abnormalities were shown in electrocardiography and echocardiography) | Low | x | low | yes |

^A^ <https://methods.cochrane.org/sites/methods.cochrane.org.prognosis/files/public/uploads/QUIPS%20tool.pdf>
^1^ Adequate description of: source population or population of interest, sampling and recruitment, period and place of recruitment, in- and exclusion criteria, study participation, baseline characteristics.
^2^ Adequate response rate, information on drop-outs and loss to follow-up, no differences between participants who completed the study and those lost to follow-up.
^3^ Method of measurement is valid, reliable, setting of measurement is the same for all participants.
^4^ Important confounders are listed (including treatments), method of measurement is valid, reliable, setting of measurement is the same for all participants, important confounders are accounted for in the design (matching, stratification, initial assembly of comparable groups), or analysis (appropriate adjustment)
^5^ Enough data are presented to assess adequacy of the analysis, strategy of model building is appropriate and based on conceptual framework, no selective reporting.

*Table of quality assessment – prognostic factor (PF) single studies*

*Based on: QUIPS^A^ (Haydn, 2006; Haydn 2013)*

| **Study reference**  (first author, year of publication) | **Study participation^1^**  Study sample represents the population of interest on key characteristics?  (high/moderate/low risk of selection bias) | **Study Attrition^2^**  Loss to follow-up not associated with key characteristics (i.e., the study data adequately represent the sample)?  (high/moderate/low risk of attrition bias) | **Prognostic factor measurement^3^**  Was the PF of interest defined and adequately measured?  (high/moderate/low risk of measurement bias related to PF) | **Outcome measurement^4^**  Was the outcome of interest defined and adequately measured?  (high/moderate/low risk of measurement bias related to outcome) | **Study confounding^5^**  Important potential confounders are appropriately accounted for?  (high/moderate/low risk of bias due to confounding) | **Statistical Analysis and Reporting^6^**  Statistical analysis appropriate for the design of the study?  (high/moderate/low risk of bias due to statistical analysis) |
| --- | --- | --- | --- | --- | --- | --- |
| Santoso, 2020 | Low risk | Moderate (information on number of patients still in the hospital when follow up ended unavailable) | Low, definition matches PICO | Low  Ic admission : moderate | High (correction for confounders not applied) | Low |
| Barman, 2020 | Low (in- and exclusion criteria defined) | Moderate (information on number of patients still in the hospital when follow up ended unavailable) | Low, definition matches PICO | Low  Ic admission : moderate | Low (correction for confounders was performed) | Low |
| Kuno, 2020 | Low | Moderate (information on number of patients still in the hospital when follow up ended unavailable) | Low, definition matches PICO | Low | High (correction for confounders not applied) | Low |
| Lorente-Ros 2020 | Moderate (matched cohort was develop but selection not transparent) | Moderate (unclear if patients that have not died or been discharged from the hospital are included in the matched cohort) | Low, definition matches PICO | Low  Ic admission : moderate | Low (correction for confounders was performed) | low |
| Wei, 2020 | Low | Moderate (information on number of patients still in the hospital when follow up ended unavailable) | Low, definition matches PICO | Low | High (correction for confounders not applied) | Moderate (for the outcome mortality the number of patients is so low that a comparison between both groups may not be appropriate) |

^A^ <https://methods.cochrane.org/sites/methods.cochrane.org.prognosis/files/public/uploads/QUIPS%20tool.pdf>
^1^ Adequate description of: source population or population of interest, sampling and recruitment, period and place of recruitment, in- and exclusion criteria, study participation, baseline characteristics.
^2^ Adequate response rate, information on drop-outs and loss to follow-up, no differences between participants who completed the study and those lost to follow-up.
^3^ Method of measurement is valid, reliable, setting of measurement is the same for all participants.
^4^ Important confounders are listed (including treatments), method of measurement is valid, reliable, setting of measurement is the same for all participants, important confounders are accounted for in the design (matching, stratification, initial assembly of comparable groups), or analysis (appropriate adjustment)
^5^ Enough data are presented to assess adequacy of the analysis, strategy of model building is appropriate and based on conceptual framework, no selective reportin
